# *Irx1* and *Irx2* Are Coordinately Expressed and Regulated by Retinoic Acid, TGFβ and FGF Signaling during Chick Hindlimb Development

**DOI:** 10.1371/journal.pone.0058549

**Published:** 2013-03-11

**Authors:** Martha Elena Díaz-Hernández, Marcia Bustamante, Claudio Iván Galván-Hernández, Jesús Chimal-Monroy

**Affiliations:** Departamento de Medicina Genómica y Toxicología Ambiental, Instituto de Investigaciones Biomédicas, Universidad Nacional Autónoma de México. México, Distrito Federal, México; Laboratoire de Biologie du Développement de Villefranche-sur-Mer, France

## Abstract

The *Iroquois* homeobox (*Irx*) genes play a crucial role in the regionalization and patterning of tissues and organs during metazoan development. The *Irx1* and *Irx2* gene expression pattern during hindlimb development has been investigated in different species, but its regulation during hindlimb morphogenesis has not been explored yet. The aim of this study was to evaluate the gene expression pattern of *Irx1* and *Irx2* as well as their regulation by important regulators of hindlimb development such as retinoic acid (RA), transforming growth factor β (TGFβ) and fibroblast growth factor (FGF) signaling during chick hindlimb development. *Irx1* and *Irx2* were coordinately expressed in the interdigital tissue, digital primordia, joints and in the boundary between cartilage and non-cartilage tissue. Down-regulation of *Irx1* and *Irx2* expression at the interdigital tissue coincided with the onset of cell death. RA was found to down-regulate their expression by a bone morphogenetic protein-independent mechanism before any evidence of cell death. Furthermore, TGFβ protein regulated *Irx1* and *Irx2* in a stage-dependent manner at the interdigital tissue, it inhibited their expression when it was administered to the interdigital tissue at developing stages before their normal down-regulation. TGFβ administered to the interdigital tissue at developing stages after normal down-regulation of *Irx1* and *Irx2* evidenced that expression of these genes marked the boundary between cartilage tissue and non-cartilage tissue. It was also found that at early stages of hindlimb development FGF signaling inhibited the expression of *Irx2*. In conclusion, the present study demonstrates that *Irx1* and *Irx2* are coordinately expressed and regulated during chick embryo hindlimb development as occurs in other species of vertebrates supporting the notion that the genomic architecture of *Irx* clusters is conserved in vertebrates.

## Introduction

In vertebrate limb development, the outgrowth and patterning of the proximo-distal axis is controlled by the apical ectodermal ridge (AER), a thickened epithelium rimming the distal tip of the growing limb, which secretes FGF to mesenchymal cells located beneath the AER, giving rise to the undifferentiated region of the limb [1], [2]. During the hand plate development, the TGFβ/Activin signaling pathway recruits undifferentiated mesenchymal cells to the cartilage lineage, prefiguring digital rays [3]. TGFβ/Activin signaling induces the formation of an ectopic digit by promoting activation of a chondrogenic molecular cascade in which *Sox9* expression is induced [4], [5], [6], [7]. In contrast, if undifferentiated mesenchymal cells are not recruited to form digital rays, they develop into interdigital tissue. In species with free digits, the loss of FGF signaling from AER promotes interdigital cell death [8], [9]. The inhibition of *Fgf8* expression in the AER is under the control of BMP signaling leading to apoptosis [9]. In addition, RA a pro-apoptotic factor, antagonizes FGF8 function inhibiting *Fgf receptor-1* gene expression [8].

The *Iroquois* genes (*Irx*) encode homeoproteins that belong to the TALE superclass of homeobox transcription factors with an atypical homeodomain and a small conserved C-terminal region in the protein: the Iroquois box [10]. In vertebrates, their genomic organization is in two cognate clusters of three genes each, cluster A includes *Irx1*, *Irx2*, and *Irx4* and cluster B, *Irx3*, *Irx5*, and *Irx6* [10]. The gene expression pattern of *Irx* genes illustrates that members of a same cluster are coordinately expressed during embryonic development [11], [12]. They have been involved in the regionalization of territories and patterning; for instance, at early stages of chick embryos, *Irx2* expression prefigures a subdivision of the neural plate [13]. In Xenopus embryos, *Iro1* is essential to specify the neural territory and dorsal mesoderm by repressing expression of *Bmp4* [14], [15].

In the hand plate development of human, mouse and chick embryos, *Irx1* and *Irx2* are expressed during digital formation [16]. In mouse limb development, *Irx1* expression takes place in the posterior digital condensation, delimited distally by the undifferentiated zone. Later, this expression expands to anterior digits, and finally it is visible in presumptive joint sites [17]. In chick limb embryo, *Irx1* expression is observed only in the digital area [16]. Meanwhile, expression of *Irx3*, *Irx5* and *Irx6* occurs in the interdigital tissue of mouse; similarly, expression of *Irx6* in chick embryos is clearly observed in the interdigital tissue [16], [18]. The mouse mutant Fused toes (*Ft*) is caused by an autosomal dominant mutation resulting from the deletion of six genes, which include cluster B of the *Irx* genes [19]. The heterozygous *Ft*/*Ft* limb phenotype is characterized by numerous and small condensations at metacarpal level, and syndactyly of fore- and hindlimbs, making the *Ft*/*Ft* mouse a useful model to study the role of *Irx* genes in limb development. In addition, the cell death pattern is altered in the anterior region of the presumptive digit area, in which misexpression of *Bmp4*, *Dkk*, *Msx1* and *Msx2* occurs [20]. On the other hand, the *Irx2* mouse mutant develops normally, and because *Irx1* and *Irx2* are coordinately expressed in the developing limb and other members of the cluster B are expressed during digit development, other *Irx* genes could possibly compensate its function.

In the present study, the main aim was to determine the gene regulation of *Irx1* and *Irx2* during chick hindlimb development. In contrast to a previous study that shows that expression of *Irx1* occurs in the digit-forming region and later in the digits, and *Irx2* expression occurs throughout the digital plate of chick embryo [16]. The present study shows that *Irx1* and *Irx2* have comparable gene expression patterns during chick hindlimb development, and that they are coordinately expressed in the interdigital tissue and digital primordia, joints and in the boundary between cartilage and non-cartilage tissue. Down-regulation of *Irx1* and *Irx2* at the interdigital tissue coincides with the onset of cell death. Furthermore, an interesting finding of this study is that RA inhibited *Irx1* and *Irx2* expression by a BMP-independent mechanism before any evidence of active caspase 3. In addition, since *Irx1* and *Irx2* expression was observed at digit primordia, this study made use of the experimental model of ectopic digit induction by TGFβ to determine the role of TGFβ in the control of *Irx1* and *Irx2* expression during digit formation in the interdigital tissue at stages before and after *Irx1* and *Irx2* down-regulation in the interdigital tissue. At developing stages before down-regulation of *Irx1* and *Irx2*, TGFβ inhibited the expression of the two genes. In contrast, at stages after down-regulation, the expression of these genes was regulated in a concentration-dependent manner. In addition, at early stages the expression of *Irx1* and *Irx2* was down-regulated by FGF signaling. Although expression of these genes has been previously reported in the development of the chick hindlimb, the present study demonstrates that *Irx1* and *Irx2* are coordinately expressed and regulated during chick embryo hindlimb development as occurs in mouse, Xenopus and zebrafish embryos lending support to the notion that the genomic architecture of *Irx* clusters is conserved in vertebrates [12].

## Results

### Down-Regulation of *Irx1* and *Irx2* in Interdigital Tissue Coincides with the Onset of Cell Death

**Figure 1:**
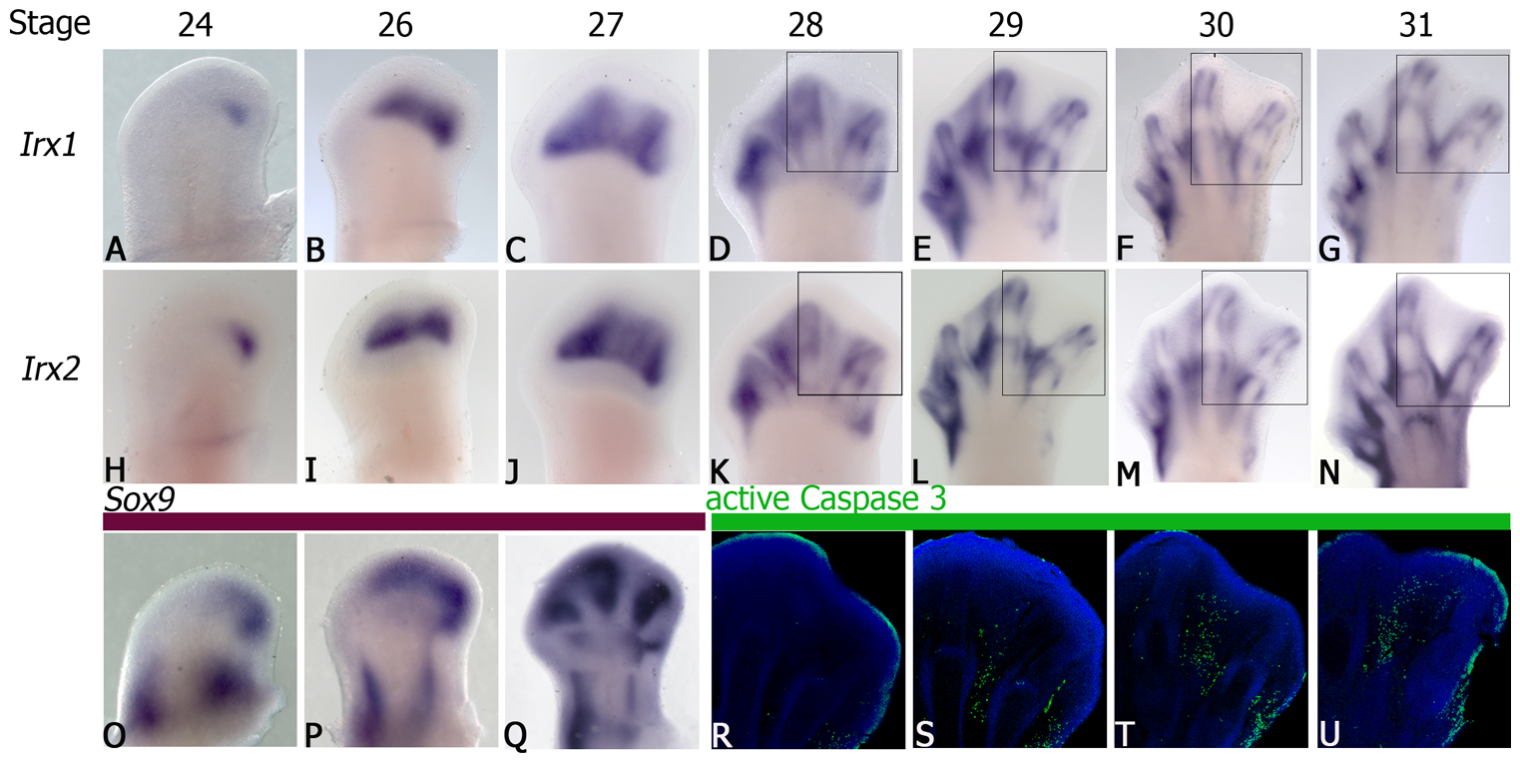
Gene expression of *Irx1* and *Irx2* is down-regulated in the interdigital tissue and coincides with the onset of cell death. Whole mount *in situ* hybridization of *Irx1* (**A**–**G**) and *Irx2* (**H**–**N**) at stages 24 HH (**A**, **H**), 26 HH (**B**, **I**), 27 HH (**C**, **J**), 28 HH (**D**, **K**), 29 HH (**E**, **L**), 30 HH (**F**, **M**) and 31 HH (**G**, **N**), respectively. Note that *Irx1* and *Irx2* are coordinately expressed in the interdigital tissue, digital primordia, joints and in the boundary between cartilage and non-cartilage tissue. *Sox9* expression at stages 24 HH (**O**), 26 HH (P) and 27 HH (**Q**) is presented to compare cartilage differentiation with *Irx* expression at developing stages 24 HH, 26 HH and 27 HH. Active Caspase 3 immunolocalization (green) in the third interdigit region of hindlimbs at 28 HH (**R**), 29 HH (**S**), 30 HH (**T**) and 31 HH (**U**) is presented to compare *Irx* interdigital expression with the cell death pattern. (**R**–**U**) Slices were cut at 50 µm. The squares indicated in D, K, E, L, F, M, G, N are to compare with panels in which the activity of caspase 3 was analyzed in R–U. Natural red color of Cy3 used to detect active caspase 3 was changed to green color to obtain a better visualization of the images.

Expression of *Irx1* and *Irx2* was evaluated during hindlimb development from stages 24–31 HH. In this study both genes were coordinately expressed at stage 24–28 HH first in the posterior region and uninterruptedly in both prospective digital and interdigital regions (Fig. 1A–D, 1H–K). Here, down-regulation of *Irx1* and *Irx2* in the third interdigit began at stage 29 HH coinciding with the onset of cell death (Fig. 1E,L,S). At stage 30–31 HH, down-regulation of both genes persisted in the first and second interdigital tissues and eventually disappeared in all interdigits and the presence of active caspase 3 is evident (Fig. 1F–G, M–N, T–U). In accordance with other reports [16], [18], *Irx1* and *Irx2* were expressed in the prospective and presumptive joint sites and in the boundary between cartilage and non-cartilage tissue.

### RA Down-Regulates *Irx1* and *Irx2* Expression Before the Onset of Cell Death

**Figure 2:**
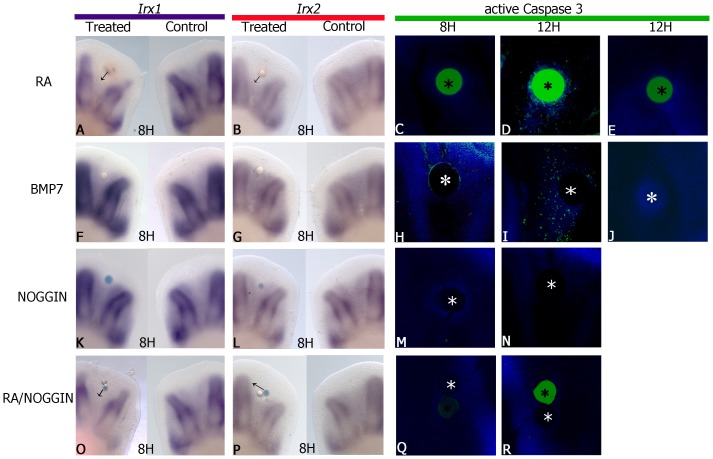
RA inhibits *Irx1* and *Irx2* expression before the onset of cell death by a BMP-independent mechanism. *Irx1* and *Irx2* expression at stage 27 HH in chick hindlimbs, after RA-treatment (**A**, **B**), BMP7-treatment (**F**, **G**), NOGGIN-treatment (**K**, **L**), and RA/NOGGIN-double treatment (**O**, **P**) for 8 h in the interdigital area. Note that *Irx1* and *Irx2* expression is inhibited by RA but not by BMP7. Active Caspase 3 (green) was evaluated in the third interdigit region of hindlimbs after RA treatment at 8 h (**C**) and 12 h (**D**) and BMP7 at 8 h (**H**) and 12 h (**I**). Note that the first signs of cell death induced by RA were observed at 12 h (**C**, **D**); however, BMP7 was able to induce the first signs of cell death at 8 h and it was most abundant at 12 h (**H**, **I**). NOGGIN-treatment (**M**, **N**), and RA/NOGGIN-double treatment (**Q**, **R**) inhibited cell death induced by RA and BMP7. Control beads without RA or BMP7 never induced cell death (**E**, **J**). Experimental samples in A, B, F, G, K, L, O, P are presented on the left; controls on the right. Black arrows indicate the area of *Irx1* and *Irx2* inhibition. Autofluorescence in green was observed in ionic-exchange beads used for RA-treatment (black asterisks). The natural red color of Cy3 used to detect active caspase 3 was changed to green for better visualization of the images. (White asterisks indicate beads soaked in BMP7 or NOGGIN).

In order to determine whether down-regulation of *Irx* genes was associated with the onset of cell death in interdigital tissue, here was evaluated the role of RA and BMP on promotion of cell death and on regulation of *Irx* genes. RA and BMPs are potent promoters of cell death during interdigital regression [21], [22], [23]. Beads soaked in the pro-apoptotic factors RA and BMP7 were placed in the third interdigit at stage 27 HH. It was observed that after 8 h, RA-treatment began to inhibit *Irx1* and *Irx2* expression in 9 out of 12 experimental cases (9/12; Fig. 2A–B). Remarkably, this inhibition occurred before the appearance of the first signs of cell death, which were first observed after 12 h of RA-treatment (3/3; Fig. 2C–D). In contrast, BMP7 or NOGGIN did not regulate *Irx1* or *Irx2* at 8 h (Fig. 2F–G, 2K–L) neither at longer treatments (data not shown). As control of functionality of the proteins, it was observed that cell death was induced by BMP7 (5/6) or inhibited by NOGGIN (7/9), at 8 and 12 h (Fig. 2H–I and 2M–N). To confirm that BMP signaling was not involved in *Irx1* and *Irx2* regulation induced by RA, one bead soaked in RA and another in NOGGIN were simultaneously placed in the third interdigit. Results showed that under these conditions NOGGIN at 8 h (Fig. 2 O–P; 6/8) or up to 24 h (data not shown) never repressed the inhibitory effect of RA on *Irx1* and *Irx2* expression, as control of functionality of the protein it was observed that cell death promoted by RA was inhibited by NOGGIN at 8 and 12 h, indicating that protein was functionally active (3/4; Fig. 2Q–R). Control beads never induced cell death (Fig. 2E, J).

**Figure 3:**
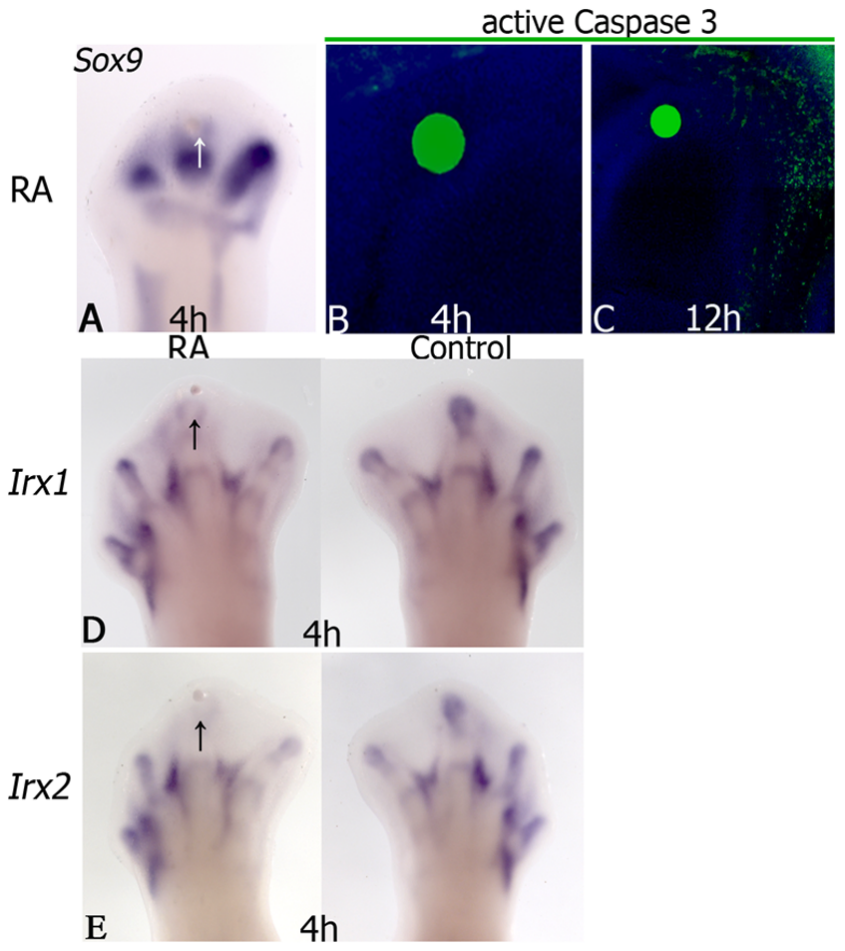
RA inhibits *Irx1* and *Irx2* expression at digit tip before the onset of cell death. *Sox9* expression in hindlimbs at stage 27 HH at 4 h (**A**) and the presence of active caspase 3 at 4 or 12 h after RA treatment in digit tip (**B**, **C**). *Irx1* and *Irx2* expression after 4 h of RA-treatment (**D**, **E**). Note the *Sox9* inhibition in digit and the presence of active caspase 3 over bead in undifferentiated region. Notice the inhibition of *Irx1* and *Irx2* expression before the presence of active caspase 3. Arrows indicate the bead position. Autofluorescence in green was observed in ionic-exchange beads used for RA-treatment. Natural red color of Cy3 used to detect active caspase 3 was changed to green color to obtain a better visualization of the images.

The expression of *Irx1* and *Irx2* was observed in skeletal primordia (Fig.1A-N) and as RA is known to inhibit chondrogenesis and promote cell death [22], the role of RA on regulation of *Irx1* and *Irx2* at the digital rays was evaluated. Results showed that *Irx1* and *Irx2* expression began to be inhibited from 4 h post-treatment (4/4) before cell death induction (4/4) that was evident from 12 h post-treatment, correlating with *Sox9* down-regulation (3/4; Fig. 3).

### TGFβ Regulates *Irx1* and *Irx2* Expression during Chondrogenesis

**Figure 4:**
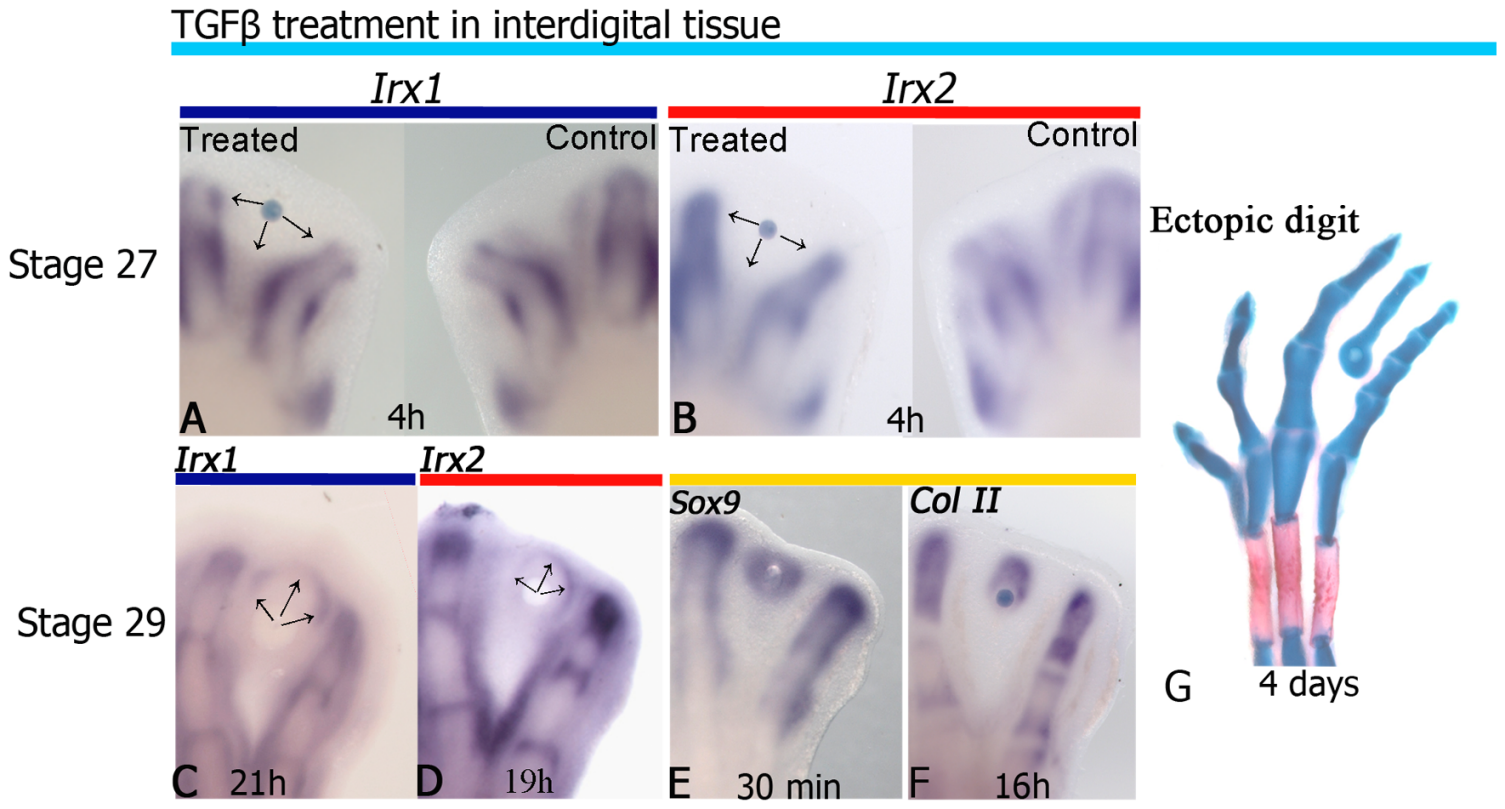
TGFβ signaling regulates *Irx1* and *Irx2* in a stage-dependent manner in the interdigital tissue. *Irx1* and *Irx2* expression after 4 h of TGFβ treatment at stage 27 HH (**A**, **B**). Regulation of *Irx1* at 21 h (**C**), *Irx2* at 19 h (**D**), *Sox9* at 30 min (**E**) and *ColII* at 16 h (**F**) after TGFβ treatment in the interdigital area at stage 29 HH. An ectopic digit is formed after 4 days of TGFβ-treatment (**G**). Note that TGFβ inhibits *Irx1* and *Irx2* expression at developing stages at which these genes are still expressed in the interdigital tissue (arrows in **A**, **B**). In contrast, TGFβ induces *Irx1* and *Irx2* expression away and around the bead (arrows in **C**, **D**) at developing stages at which *Irx1* and *Irx2* expression has been down-regulated in the interdigital tissue.

On the basis that RA and TGFβ have antagonistic functions in the control of chondrogenesis and cell death, and that in the present study the concomitant down-regulation of *Irx1*, *Irx2* and *Sox9* expression occurred after RA treatment, the role of TGFβ in the regulation of *Irx1* and *Irx2* was evaluated at the interdigital tissue during ectopic digit formation [5], [7], [23] by performing two experiments. The first experiment examined if TGFβ regulated *Irx1* and *Irx2* expression at developmental stages before normal down-regulation of these genes in the interdigital tissue. Thus, beads soaked in TGFβ were placed in the interdigital tissue of hindlimbs at stage 27 HH. Results showed that inhibition of *Irx1* and *Irx2* expression began at 4 h of TGFβ treatment (10/10; Fig. 4A–B). Similarly, the second experiment was performed in ectopic digit at 29 HH to explore if TGFβ was able to regulate *Irx1* and *Irx2* expression, which normally disappeared from the interdigital tissue. Results showed that *Irx1* expression began after 21 h (4/5) of TGFβ treatment, while *Irx2* did so at 19 h (6/8; Fig. 4C–D). Remarkably, the *Irx1* and *Irx2* expression pattern induced by TGFβ seemed to be limited away and around the bead resembling a perichondrium as compared with *Sox9* expression (4/4; Fig. 4C–E). To ensure that the formation of the ectopic digit in the interdigital tissue is due to TGFβ, gene expression of *Sox9* and *ColII* was evaluated [7]. Results confirmed that TGFβ-treatment triggers the chondrogenesis molecular cascade because it was able to induce the onset of *Sox9* expression and *ColII*, 30 min and 16 h after treatment, respectively, and ended in the formation of an ectopic digit (6/8; Fig. 4 E–G) as previously reported [7]. In our hands, the implantation of beads soaked in PBS never gave rise to ectopic digits (data not shown), suggesting that simply wounding into interdigital tissue did not trigger their formation.

**Figure 5:**
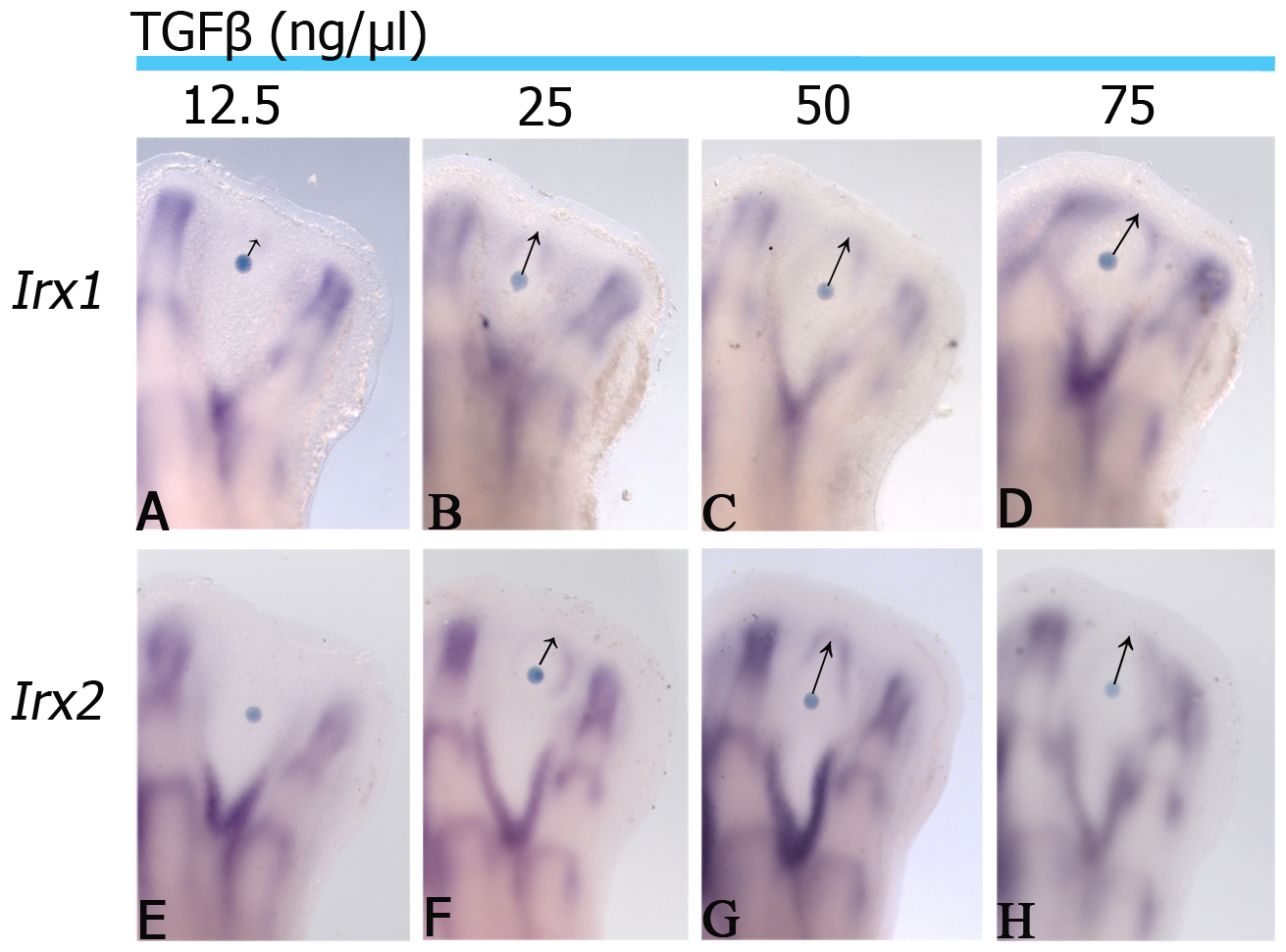
*Irx1* and *Irx2* expression is regulated in a concentration-dependent manner by TGFβ. *Irx1* and *Irx2* expression at stage 29 HH in chick hindlimbs after 21 hours of TGFβ treatment at different doses 12.5 ng/µl (**A**, **E**) 25 ng/µl (**B**, **F**) 50 ng/µl (**C**,**G**) 75 ng/µl (**D**, **H**), respectively in the interdigital area. The black arrows show the distance of *Irx1* and *Irx2* induction.

**Figure 6:**
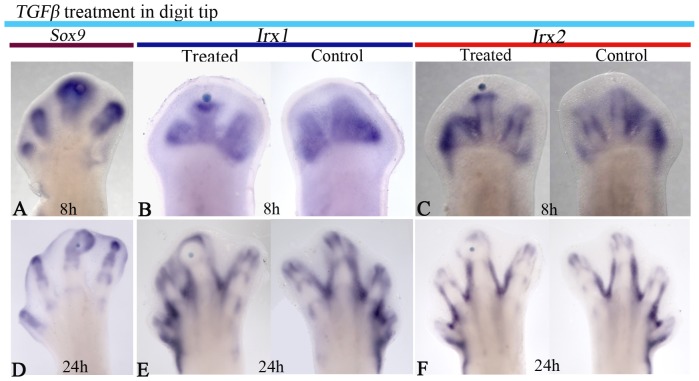
TGFβ regulates *Irx1* and *Irx2* expression in digit tip. *Sox9* expression at 8 h (**A**) and 24 h (**D**); *Irx* and *Irx2* expression after 8 h (**B**, **C**) of TGFβ treatment in the digit tip at stage 27 HH. Note that TGFβ inhibits *Irx1* and *Irx2* expression over the bead, while below, their expression was noted as a transversal line in the phalanx. *Irx1* and *Irx2* expression after 24 h of TGFβ-treatment (**E**–**F**). Note that *Irx1* and *Irx2* are expressed around the enlarged and rounded cartilage and compare with (**D**) which shows *Sox9* expression at the digit tip after 24 h of TGFβ-treatment.

As the *Irx1* and *Irx2* expression pattern observed in the interdigital tissue is inhibited or induced around cartilage condensations by TGFβ depending on the developmental stage, and they are expressed at the edge of skeletal elements, it is possible to speculate that *Irx1* and *Irx2* expression is established once the wavefront of cartilage differentiation is finished. To test this hypothesis, beads soaked in TGFβ at different concentrations were placed at the interdigital tissue, and after 21 h it was observed that expression of *Irx* genes was established further away from the bead, in a concentration-dependent manner, although similar results were obtained at 50 and 75 ng/µl TGFβ (Fig. 5). On this basis, the following experiment was done to promote chondrogenesis on the tip of digits and then evaluate whether *Irx* genes would be expressed at the boundary between the new cartilage at the tip of digit and non-cartilage tissue. Beads soaked in 50 ng/µl TGFβ were placed at the tip of digits at stage 27 HH. We preferred to use this concentration because it had similar effects that 75 ng/µl TGFβ. Under these conditions, the expression of *Sox9* in the phalanx was enlarged showing a rounded appearance (3/4; Fig. 6A). *Irx1* and *Irx2* expression seemed inhibited over the bead from 8 h, while below it the expression was noted as a transversal line in the phalanx (Fig. 6A–C). After 24 h of TGFβ-treatment, *Irx1* and *Irx2* expression appeared around the enlarged and rounded cartilage (6/9) that is *Sox9* positive, resembling a perichondrium (Fig. 6D–F).

### *Irx1* and *Irx2* Expression is Regulated by FGF Signaling

**Figure 7:**
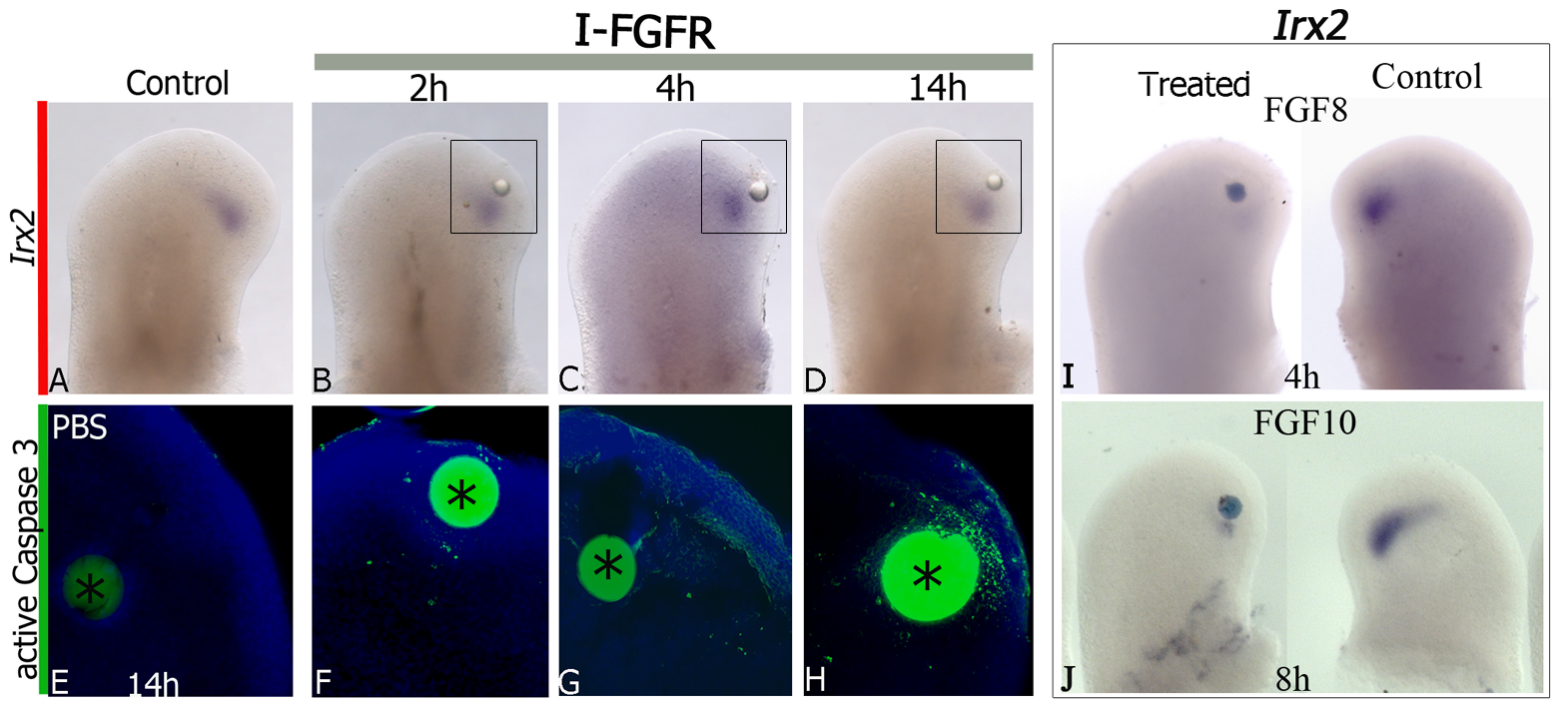
FGF Regulates *Irx2* expression. *Irx2* expression (**A**–**D**) and the presence of active caspase 3, after 2 h (**F**), 4 h (**G**) and 14 h (**H**) of FGFR-inhibitor (I-FGFR) SU5402 treatment. The squares indicated in B–D are to compare with panels in which the activity of caspase 3 was analyzed in E–H. Control bead in PBS did not promote cell death (**E**). Note that inhibition of FGF signaling did not affect *Irx2* expression but promoted cell death from 2 h. *Irx2* expression after 4 h of FGF8 treatment at stage 24 HH (**I**) and FGF10 treatment at 8 h (**J**) in hindlimb posterior region. Note that inhibition of *Irx2* expression by action of FGF8 occurred earlier that FGF10.

An observation obtained from the *Irx1* and *Irx2* gene expression pattern is that they were not expressed in the undifferentiated zone beneath AER. It is known that this zone is dependent on FGF signaling. Because in this study *Irx1* and *Irx2* are coordinately expressed and regulated by RA and TGFβ, we solely evaluated the expression of *Irx2* in the undifferentiated zone beneath AER region to determine whether non-expression of *Irx2* is a consequence of inhibition by FGF signaling. Beads soaked in the FGFR-selective inhibitor SU5402 were placed in the undifferentiated zone beneath AER of hindlimbs at stage 24 HH. This treatment was not able to induce *Irx2* expression at 2, 4 h, or later, instead it resulted in activation of cell death that was evident at low level from 2 h but it extended from 4 h (Fig 7A–H). Because this analysis did not allow to determine whether the inhibition of FGF signaling regulate *Irx2* expression, we determined whether beads soaked in FGF8 or FGF10, placed in posterior-distal region of hindlimbs at stage 24 HH, have effects on *Irx2* expression. Results showed that its expression began to be inhibited by FGF8 (3/3) and FGF10 (4/4) from 4 h and 8 h, respectively (Fig. 7I–J).

## Discussion

Findings reported by McDonald et al [16] show the expression of *Irx1* present in the digit-forming region and later in the digits, while *Irx2* is highly expressed throughout the digital plate of the chick embryo. In contrast with that study, the present study showed that *Irx1* and *Irx2* were coordinately expressed during hindlimb development, as shown in other animal models [12], [18]. The two genes were expressed as a continuous label comprising well-defined areas of mesodermal tissue of both prospective digital and interdigital areas, and later in digital and interdigital regions. In fact, the *Irx2* probe used in the present study was also used in the mentioned report [16]. Probably, the high concentrations of proteinase K and the elimination of the Iro and homeo box from both probes allowed us to obtain a specific expression of both genes in hindlimb development.

**Figure 8:**
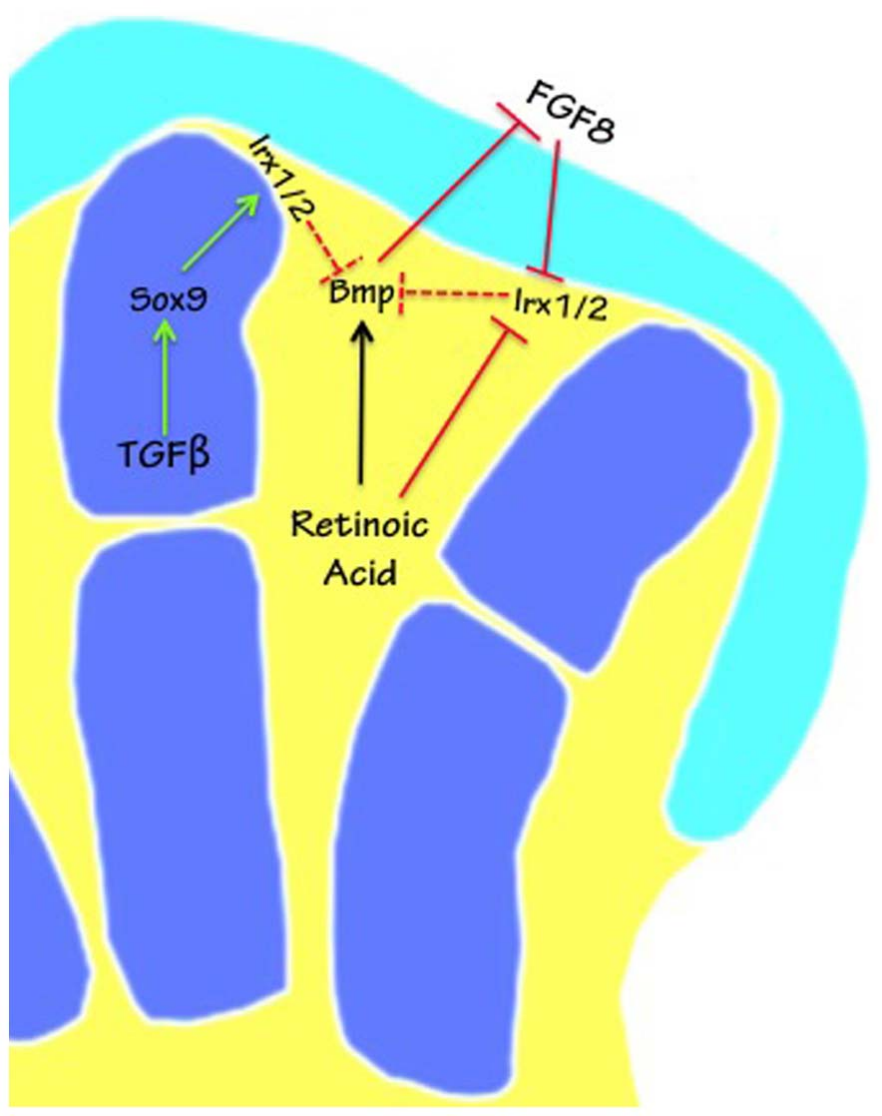
Model of interactions showing *Irx* regulation by RA, TGFβ and FGF. Schematic representation of the control of *Irx1* and *Irx2* (*Irx1*/2) expression in digit regions (blue) versus interdigit regions (yellow). White lines delineating the digits represent the expression of *Irx1*/*2* in the perichondrium. Dotted lines represent hypothetical inhibition of the IRX protein by *Bmp4*, and perhaps *Bmp7* and *Msx* (not shown) based on reports in the literature [14], [15], [26]. *Fgf10* is not indicated, but it promotes *Fgf8* expression [37]. Cyan represents the undifferentiated region of the limb.

Here was observed that down-regulation of *Irx1* and *Irx2* expression in interdigital tissue coincided with the commencement of cell death. However, an interesting finding is that in the interdigital tissue, RA inhibited *Irx1* and *Irx2* expression before the first signs of active caspase 3 were observed. The role of RA in programmed cell death control during hindlimb development is well known, as well as its effects mediated by BMP signaling [8], [22], [24], [25]. In the present study, RA inhibited *Irx1* and *Irx2* expression by a BMP-independent mechanism. On this basis, the present study suggests that *Irx1* and *Irx2* expression may play a protective role against cell death (Fig. 8). It follows that down-regulation of *Irx* genes in the interdigital tissue by RA may be a pre-requisite to promote the molecular cascade that ends in cell death, since *Irx* genes may repress the expression of genes involved in the cell death process, as has been suggested for the *Msx* gene in the boundary between body wall and wing in *Drosophila*, and for *Bmp4* during *Xenopus* development [14], [15], [20], [26]. This protector effect of *Irx* against cell death is probably lost in the limbs of fused-toes mutant mice, which show massive cell death and up-regulation of *Bmp4* and *Dkk1*, as well as down-regulation of *Fgf8* and *Fgf10* [20]. Interestingly, *Irx3*, *Irx5* and *Irx6* are expressed in the interdigital tissue of the mouse limb as occurs for the *Irx1* and *Irx2* genes in the chick embryo. So, it is possible that *Irx* genes may be involved in cell death control. Supporting this hypothesis, in other systems the deletion of *Irx* genes results in cell death, for instance, *Irx1a*, *Irx4a* or *Irxl1* morphants show extensive cell death in the rostral region of the zebrafish embryo [27], in the CNS [28] and in the developing head and trunk along the neural tube [29], respectively. Moreover, antisense knockdown of pulmonary *Irx1*, *Irx2*, *Irx3*, and *Irx5* together increases apoptosis in the mesenchymal compartment of rat lung explants [30].

In this study, the expression of *Irx1* and *Irx2* was observed in the boundary of skeletal primordia and non-cartilage tissue and in these regions RA also inhibited *Irx1* and *Irx2* expression concomitantly with down-regulation of *Sox9*, before induction of cell death. Because it is known that RA is an important inhibitor of chondrogenesis [22], and on the basis that the products of the *Irx* genes repress *Bmp4* or *Msx* expression in other systems, two genes involved in cell death during limb development [14], [15], [20], [26], it may be considered that *Irx* gene expression during digital formation protects cells committed to the chondrocyte lineage against cell death-promoting signals, delimiting the boundaries of skeletal elements (Fig. 8). It is important to mention that the temporal gene expression of *Irx* genes (around 21 h) and *Sox9* (at 30 min) during ectopic digit formation induced by TGFβ, and the concomitant down-regulation of *Irx1*, *Irx2* and *Sox9* expression, which occurred after the first 4 h of RA treatment, suggest that *Irx* genes are not needed for chondrogenesis.

On the other hand, depending on the developmental stage, TGFβ was also able to regulate *Irx1* and *Irx2* expression in the interdigital tissue. At developing stages before down-regulation of *Irx1* and *Irx2*, TGFβ produced a halo of inhibition. Whereas, at developing stages after their down-regulation, *Irx1* and *Irx2* expression appeared to be limited to areas away from the bead and around the expression of *Sox9* forming a boundary between non-cartilage tissue and cartilage. Interestingly, it was demonstrated herein that these effects of TGFβ on *Irx* gene expression are concentration-dependent. Moreover, when TGFβ was placed at the tip of the digits, after the first 8 h of treatment the expression of *Irx1* and *Irx2* was observed as a transverse line that disappeared after 24 h of treatment. This may reflect that a first wavefront of *Irx* gene expression inhibition by TGFβ is working, as observed in the interdigital tissue at stages at which *Irx* genes are present in this tissue. Later, a wavefront of differentiation induced by TGFβ may restrict *Irx* gene expression to the edges of cartilage. On this basis, it is reasonable to propose that expression of *Irx1* and *Irx2* during digit development delimits the boundary between cartilage tissue and non-cartilage tissue. The establishment of this boundary may reflect the range of diffusion of TGFβ to promote chondrogenesis, which may be counteracted by a chondrogenesis inhibitor, as suggested elsewhere [31], [32], [33]. Another interpretation is that *Irx1* and *Irx2* expression at this site could depend on a threshold [34], presumably of TGFβ. These interactions may conceivably allow the formation of the prospective perichondrium.

Another interesting finding was that the *Irx1* and *Irx2* gene expression patterns were excluded from the undifferentiated zone beneath AER. Furthermore, it was not possible to determine the regulation of *Irx2* expression by inhibition of FGF signaling because cell death was induced from 2 h and at later times it was more extended, making difficult to determine the regulation of *Irx2* by FGF signaling. However, the treatment with FGF proteins indicated that *Irx2* expression began to be inhibited by FGF8 and FGF10 from 4 h and 8 h, respectively. On the other hand, it has previously been demonstrated that during limb development FGF10 induces expression of FGF8, while FGF8 is not able to induce FGF10 [35]. On this basis it is reasonable to speculate that *Irx2* gene expression pattern is excluded from the undifferentiated zone beneath AER by action of FGF8 that inhibits expression of *Irx2*, while inhibition by FGF10 observed in this study may be mediated by FGF8 (Fig. 8).

Finally, this work shows that *Irx1* and *Irx2* were coordinately expressed and regulated by RA, TGFβ and FGF signaling during chick hindlimb development. Tena et al [12] suggested that coordinate regulation of members of the *Iroquois* family in mouse, Xenopus and zebrafish is under the control of a genomic architecture. This generates a 3D conformation that allows the promoters of the genes of each cluster are to be located in close proximity [12]. These authors suggest the establishment of regulatory landscapes for certain genes of the cluster in a tissue- and stage-dependent manner [12]. The demonstration in the current study that the coordinated regulation of *Irx1*and *Irx2* expression by RA, TGFβ and FGF signaling during hindlimb development in chick embryos, supports the notion that the genomic architecture of *Irx* clusters is conserved in vertebrates [12].

## Materials and Methods

### Ethics Statement

This research protocol was reviewed and approved by the Institutional Review Board for the Care and Use of Laboratory Animals of Instituto de Investigaciones Biomédicas, UNAM.

### Eggs and Embryo Manipulations

Fertilized White Leghorn chicken eggs (ALPES, Puebla, Mexico) were incubated at 38°C and the embryos staged according to Hamburger and Hamilton (HH) [36]. To manipulate embryos, the fertilized eggs were windowed at stage 27–29 HH and the right leg bud was exposed for experimentation. Affigel (Bio-Rad Laboratories, Hercules, CA) and heparin acrylic beads (Sigma-Aldrich, St. Louis, MO, USA) were soaked in the following human recombinant proteins: 1 mg/ml for FGF8, FGF10 or NOGGIN; 2 mg/ml BMP-7, 12.5, 25, 50 and 75 ng/µl TGFβ (Peprotech, Mexico City, Mexico). AGI-X2 ionic exchange beads (Sigma-Aldrich) were soaked in 5 mg/ml RA (Sigma-Aldrich) or 20 mM SU5402 (Calbiochem, Billerica, MA, USA). Treatments were done in interdigital tissue, digit tips or posterior region at stages from 24 HH to 29 HH; later, eggs were returned to the incubator. Embryo analysis was done at different times by whole-mount *in situ* hybridization.

### RT-PCR, cDNA Probes and *In Situ* Hybridization

The following cDNA probes were used for *in situ* hybridization; *Sox9* and *ColII* described in [7]. An *Irx1* probe was obtained by RT-PCR. In brief, RNA was isolated from chick hindlimb buds at stages 26–30 HH using TRIZOL reagent (Invitrogen, Carlsbad, CA, USA). Total cDNA was obtained by reverse transcription with the First Strand cDNA Synthesis Kit for RT-PCR (AMV) using oligo (dT) primer according to the manufacturer's protocol (Roche Applied Science, Indianapolis, IN, USA). The PCR was done using the following primers for *Irx1* (accession number AJ238354), forward primer 5′-GCTCAATGAACACCGCAAG-3′ and reverse 5′-GTTGTGGTGAGTGGCATGGT-3′. A fragment of 757 bp was cloned into the pGEM vector using a T-easy pGEM Vector System I (Promega, Madison, WI, USA). The *Irx2* fragment has been previously described [13]. To ensure the specificity of *Irx1* and *Irx2* probes, the homeobox and Iro box were deleted from both probes, and the sequences of both cDNA were compared with those of other members of the *Iroquois* gene family; none were found to match. The RNA anti-sense probes were labeled with UTP-digoxigenin (Roche) and used for whole-mount *in situ* hybridization as described previously [37]. Samples were treated with 60 µg/ml proteinase K for 25 min at 21°C for *Irx1*, *Irx2* and *Col II*; and for 28 min at 25°C for *Sox9*. The hybridization temperature was 68°C and post-hybridization washes were done at 70°C. Reactions were visualized with BM purple substrate for alkaline phosphatase (Roche Applied Science).

### Cartilage Staining

For skeletal staining, the hindlimbs were washed in PBS (Phosphate buffer saline) for 10 min, fixed in ethanol followed by acetone and stained in a solution of 0.3% Alcian blue/0.1% Alizarin red for 24 h each. Samples were washed twice for 10 min in water, and then treated with a solution of 1% KOH/20% glycerol. Stained cartilage hindlimbs were stored in 50% glycerol/50% ethanol for photography.

### Immunodetection of Active Caspase 3

To detect cell death, caspase 3 activation was evaluated in 50 or 100 µm tissue sections of hindlimbs previously fixed and cryoprotected in 30% sucrose. Tissue sections were incubated with active caspase 3 antibody (1∶200; Abcam, Cambridge, UK). Samples were washed with 0.3% Triton in PBS and incubated with Cy3 secondary antibody (1∶500, Jackson Laboratory, Sacramento, CA, USA) for 2 h at room temperature. Finally, cell nuclei were stained with 1 mg/ml 4′6′-diamidino-2-phenylindole dihydrochloride (DAPI) (Sigma-Aldrich) and samples mounted with Dako Fluorescence Mounting Medium (Dako, USA). Images were obtained with a Hamamatsu C9100 CCD in an Olympus BX51 WI microscope interfaced with StereoInvestigator Software 9.1.4.5 (MBF Bioscience, Williston, USA).
